# Supplementary material for: A de novo matrix for macroscopic living materials from bacteria
Source: Nat Commun. 2022 Sep 21;13:5544. doi: 10.1038/s41467-022-33191-2 (PMC9492681; doi:10.1038/s41467-022-33191-2)
Supplement: Supplementary file 1 — Supplementary Information [file 41467_2022_33191_MOESM1_ESM.docx]

**A *de novo* matrix for macroscopic living materials from bacteria**

Sara Molinari^1^, Robert F. Tesoriero^1,2^, Dong Li^3^, Swetha Sridhar^1,2^, Rong Cai^1^, Jayashree Soman^1^, Kathleen R. Ryan^4^, Paul D. Ashby^3^, Caroline M. Ajo-Franklin^1,*^.

Correspondence to: [cajo-franklin@rice.edu](mailto:cajo-franklin@rice.edu)

**Fig. S1. The wild-type strain does not form macroscopic aggregates.** Representative image of wild-type *C. crescentus* strain (Mfm126) grown under standard conditions. The image was taken from the bottom of a 250 mL flask and shows the typical turbidity of a saturated culture of *C. crescentus* with no visible cellular aggregates. The scale bar is 1 cm.

**Fig. S2. The *∆SpyTag* strain does not bind SpyCatcher-GFP.** (**a**) Genetic constructs replacing the native *rsaA* gene in the *∆SpyTag* strain (left panel). Image of corresponding *∆SpyTag* BUD-ELM (right panel) which is indistinguishable from the original BUD-ELM. Scale bar is 1 cm. (**b**) Representative image of single cells of the *∆SpyTag* strain (yellow channel, mKate2) incubated with SpyCatcher-GFP (cyan channel, GFP), showing no significant staining. In every image the scale bar is 10 µm.

**Fig. S3. AFM images show cell-cell interactions in the BUD-ELM strain.** Representative AFM images at different magnifications of single cells of the BUD-ELM strain, revealing the presence of cell-cell interactions. The junctions of interacting cells show soft material accumulation. BUD-ELM cells also appear with the characteristic brush-like structure of the cell surface.

**Fig. S4. The secreted matrix within BUD-ELMs is surrounded by cells.** Representative confocal microscopy stack images of the same BUD-ELM section. They show that the protein matrix, stained with Congo Red, is fully enclosed by cells. Scale bar is 10 µm and applies to every image.

**Fig. S5. BUD-ELMs contain a proteinaceous matrix.** Confocal microscopy of BUD-ELMs stained with Congo Red (left panel) and DiO (right panel), highlighting the proteinaceous nature of the BUD-ELM matrix. Scale bars are 10 µm and apply to every image.

**Fig. S6. The anti-FLAG antibody does not stain any protein of the wild-type culture supernatant.** The image shows an anti-FLAG® immunoblot of supernatants of the wild-type (lanes 2-4, three independent replicates) and BUD-ELM (lanes 5-7, three independent replicates) cultures. Lane 1 and 8 show the protein ladder. This immunoblot confirms that the FLAG-tag does not bind to any protein of the wild-type supernatant, confirming previous literature data^29^. Source data are provided as a Source Data file.

**Fig. S7. Cells of the *∆ELP_60_* BUD-ELM strain are surrounded by an external layer of BUD protein.**Confocal microscopy of single cells of BUD-ELM strain after staining with SpyCatcher-GFP (left) or GFP (right), demonstrating that the *∆ELP_60_* BUD protein is located on the cell surface and a layer of proteinaceous matrix encapsulate every cell, similarly to what we observed in the original BUD-ELM strain. Scale bar is 5 µm and applies to every image.

**Fig. S8. The *∆ELP_60_* BUD-ELM strain displays more BUD protein than the original BUD-ELM strain.**Anti-FLAG® immunoblot of whole cell lysates of the BUD-ELM (lanes 2-4, three independent replicates) and *∆ELP_60_* BUD-ELM (lanes 5-7, three independent replicates). Lane 1 shows the protein ladder. This immunoblot corroborates the observed molecular weight of the BUD protein from Fig. S6 and Fig. 2e. Note that also ∆ELP_60_ BUD protein (62.8 kDa) appears to run at the higher molecular weight (~75 kDa), in accordance with prior observations on the native RsaA^30^ and the original BUD protein. Source data are provided as a Source Data file.

**Fig. S9. Disruption of the liquid-air interface, through antifoam addition, prevents the formation of BUD-ELMs. a**) BUD-ELM strain grown under standard conditions with the addition of 0.02% antifoam show no visible pellicle formation at the air-water interface and consequently **b**) no material formation.

**
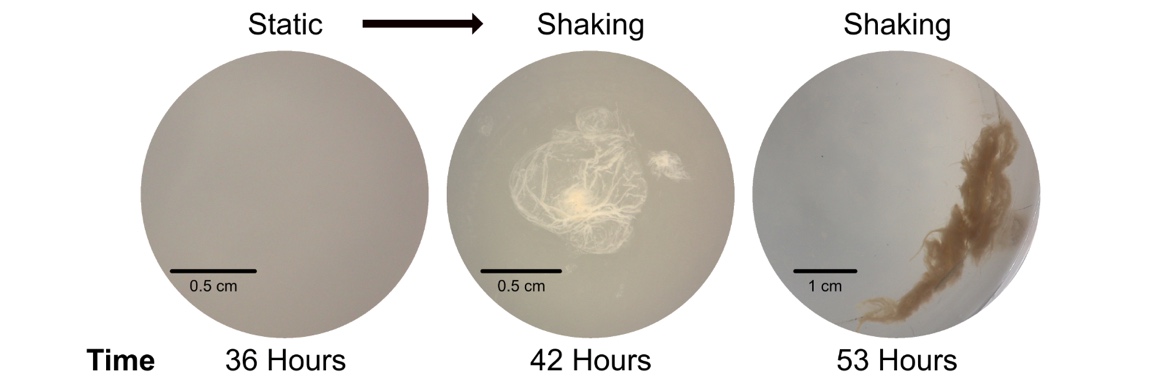
**

**Fig. S10. Static-Grown Cultures form BUD-ELM in response to shaking.**BUD-ELM strain grown in static conditions showed no material formation after 36 h (left image). When shaking was applied to cultures, pellicle (middle image) and final material (right image) formation were observed within 6 and 11 h, respectively. Scale bars are 0.5 cm for the left and middle image and 1 cm for the right image.

**Fig. S11. Relationship between the major components of the Modified Volumetric Power and Material Size. a**) Flat surface area of BUD-ELMs was plotted against the Volumetric Power input (left), k_L_a (middle), and the product of the two (right). None of these relationships provide a consistent metric for predicting large BUD-ELM size across flask sizes. Error bars are centered on the mean value and represent the standard error of the mean at least three samples. X-axis label and legend apply to every graph. **b**) Correlation between modified volumetric power and the apparent surface area of the BUD-ELMs grown in 125 mL and 250 mL shake flasks. Error bars are centered on the mean value and represent the standard error of the mean of at least three independent samples. Source data are provided as a Source Data file.

**Fig. S12. Oscillatory strain sweep**. Strain sweep measurements were acquired from 0.1% to 100% strain amplitude at a constant frequency of 3.14 rad/s. Error bars are centered on the mean value and represent 95% confidence intervals of at least five samples. From the amplitude sweep curves, we identified the linear viscoelastic region of the three BUD-ELMs and set the strain used to collect frequency sweep data (Fig. S13) to 0.35%. Source data are provided as a Source Data file.

**Fig. S13. Frequency sweep curve.**Frequency sweep measurements were acquired from 0.1 rad/s to 100 rad/s at a constant strain amplitude of 0.35%. Error bars are centered on the mean value and represent 95% confidence intervals of at least five samples. Source data are provided as a Source Data file.

**Fig. S14. Reaction rate of the cell lysate extracted from strains producing SpyCatcher-holo-GDH and holo-GDH.** (**a**) holo-GDH show enzymatic activity in cell lysates of *E. coli* strains expressing GDH (top) or SpyCatcher-GDH (bottom). Error bars are centered on the mean value and represent standard deviation of three independent samples. (**b**) Kinetic curve of the enzymatic conversion of 2,6-dichlorophenol–indophenol (DCPIP) by BUD-ELM incubated with SpyCatcher-holo-GDH (circles) or holo-GDH (squares). Error bars are centered on the mean value and represent standard errors of three independent samples. Source data are provided as a Source Data file.

| **Strain Name** | **Strain Designation** | **Source** | **genotype** |
| --- | --- | --- | --- |
| wild type | MFm126 | A. Persat *et al*^47^ | NA1000 *ΔsapA :: Pxyl mKate2* |
| ∆*rsaA* | MFm133 | M. Charrier *et al*^31^ | NA1000 *ΔrsaA:: Spec* |
| ∆*rsaA_1-250_* | MFm152 | M. T. Orozco-Hidalgo *et al*^29^ | NA1000 *ΔsapA::Pxyl-mKate2,*  *ΔrsaA(1-689):: ELP_60_* |
| original  BUD-ELM | RCC002 | This work | NA1000 Δ*sapA::Pxyl mKate2,* *ΔrsaA(251-689)::* *ELP_60_-SpyTag* |
| *∆ELP_60_*  BUD-ELM | RCC004 | This work | NA1000 Δ*sapA::Pxyl mKate2,* *ΔrsaA(251-689) ::* *SpyTag* |
| ∆*SpyTag*  BUD-ELM | RCC005 | This work | NA1000 Δ*sapA::Pxyl mKate2,* *ΔrsaA(251-689):: ELP_60_* |

**Table S1. List of *C. crescentus* strains used in this work.**

| **Plasmid** | **Source** | **Description** |
| --- | --- | --- |
| pSMCAF008 | This work | Integration plasmid for strain RCC002. *rsaA-N-terminus* (800bp), *(ELP)_60_, SpyTag, rsaA-C-terminus* (800bp) |
| pSMCAF017 | This work | Integration plasmid for strain RCC004. *rsaA-N-terminus* (800bp), *SpyTag, rsaA-C-terminus* (800bp) |
| pSMCAF018 | This work | Integration plasmid for strain RCC005. *rsaA-N-terminus* (800bp), *(ELP)_60_, rsaA-C-terminus* (800bp) |
| pSMCAF015 | This work | *pAra, HisTag-Tev-GFP*; L-arabinose-induced expression of GFP, used for protein purification. |
| pSMCAF016 | This work | *pAra, HisTag-Tev-SpyCatcher-GFP*; L-arabinose-induced expression of SpyCatcher-GFP, used for protein purification. |
| pSMCAF029 | This work | *pAra, HisTag-Tev-SpyCatcher-GDH*; L-arabinose-induced expression of SpyCatcher-GDH, used for BUD-ELM functionalization. |
| pSMCAF032 | This work | *pAra, HisTag-Tev-GDH*; L-arabinose-induced expression of GDH, used for BUD-ELM functionalization. |
| KR-12 | Provided by Prof. Kathleen R. Ryan | *pTac, GFP-mut3*. IPTG-induced expression of GFP-mut3 in *C. crescentus*. (Fig. 2B – Congo Red staining). |

**Table S2. List of plasmids used in this work.**

| **Flask Volume (mL)** | **Shaking Speed (rpm)** | **Culture Volume (mL)** | **Modified Volumetric Power (mW∙m^2^/s)** |
| --- | --- | --- | --- |
| 125 | 150 | 25 | 0.166038 |
| 125 | 150 | 30 | 0.125036 |
| 125 | 200 | 30 | 0.360296 |
| 125 | 225 | 25 | 0.738597 |
| 125 | 225 | 30 | 0.556207 |
| 125 | 250 | 25 | 1.08964 |
| 125 | 250 | 30 | 0.820563 |
| 250 | 150 | 50 | 0.518068 |
| 250 | 150 | 60 | 0.390136 |
| 250 | 150 | 80 | 0.249383 |
| 250 | 200 | 60 | 1.129326 |
| 250 | 200 | 80 | 0.721889 |
| 250 | 225 | 50 | 2.319188 |
| 250 | 225 | 60 | 1.746486 |
| 250 | 225 | 80 | 1.11639 |
| 250 | 250 | 30 | 7.585363 |
| 250 | 250 | 50 | 3.426718 |
| 250 | 250 | 60 | 2.580522 |
| 250 | 250 | 70 | 2.030337 |
| 250 | 250 | 75 | 1.823722 |
| 250 | 250 | 80 | 1.649523 |

**Table S3. Growth conditions used to generate the model in Fig. 3D.** Flask type, shaking speed, culture volume, and modified volumetric power of cultures used to grow the BUD-ELMs used to build the model in Fig. 3D.

| **Shaking Speed (rpm)** | **Culture Volume (mL)** | **Modified Volumetric Power (mW∙m^2^/s)** | **Predicted size regime** | **Resulting Size regime** | **Resulting Size (cm^2^)** |
| --- | --- | --- | --- | --- | --- |
| 100 | 120 | 0.40 | Small | Small | 0.033516 ± 0.015 |
| 150 | 160 | 1.15 | Large | Large | 0.632177 ± 0.04 |
| 150 | 120 | 1.80 | Small | Small | 0.077027 ± 0.012 |
| 216 | 160 | 4.45 | Small | Small | 0.070895 ± 0.03 |

**Table S4. Growth conditions, predicted and observed parameters used to test the model in Fig. 3D.** These values refer to cultures in 500 mL flasks. All error is measured in terms of standard error.

**Source Data Files**

**Fig. S6:** uncropped blot.

**Fig. S8**: uncropped blot
