# Supplementary material for: A de novo matrix for macroscopic living materials from bacteria
Source: Nat Commun. 2022 Sep 21;13:5544. doi: 10.1038/s41467-022-33191-2 (PMC9492681; doi:10.1038/s41467-022-33191-2)
Supplement: Supplementary file 2 — Reporting Summary [file 41467_2022_33191_MOESM2_ESM.pdf]

nature portfolio

Corresponding author(s): Caroline Ajo-Franklin

Last updated by author(s): 08/25/2022

Reporting Summary

Nature Portfolio wishes to improve the reproducibility of the work that we publish. This form provides structure for consistency and transparency in reporting. For further information on Nature Portfolio policies, see our Editorial Policies and the Editorial Policy Checklist.

Statistics

For all statistical analyses, confirm that the following items are present in the figure legend, table legend, main text, or Methods section.

n/a	Confirmed	
☐	☒	The exact sample size (*n*) for each experimental group/condition, given as a discrete number and unit of measurement
☐	☒	A statement on whether measurements were taken from distinct samples or whether the same sample was measured repeatedly
☒	☐	The statistical test(s) used AND whether they are one- or two-sided<br>*Only common tests should be described solely by name; describe more complex techniques in the Methods section.*
☒	☐	A description of all covariates tested
☒	☐	A description of any assumptions or corrections, such as tests of normality and adjustment for multiple comparisons
☐	☒	A full description of the statistical parameters including central tendency (e.g. means) or other basic estimates (e.g. regression coefficient) AND variation (e.g. standard deviation) or associated estimates of uncertainty (e.g. confidence intervals)
☒	☐	For null hypothesis testing, the test statistic (e.g. *F*, *t*, *r*) with confidence intervals, effect sizes, degrees of freedom and *P* value noted<br>*Give P values as exact values whenever suitable.*
☒	☐	For Bayesian analysis, information on the choice of priors and Markov chain Monte Carlo settings
☒	☐	For hierarchical and complex designs, identification of the appropriate level for tests and full reporting of outcomes
☒	☐	Estimates of effect sizes (e.g. Cohen's *d*, Pearson's *r*), indicating how they were calculated

Our web collection on statistics for biologists contains articles on many of the points above.

Software and code

Policy information about availability of computer code

Data collection	Rheological data was acquired using TRIOS (version 4.2.1.36612) ; Confocal microscopy data was collected using the ZEN (version 2.6) software; Optical microscopy data was acquired using the software NIS-Elements AR (version 4.51.01);
Data analysis	Cadium Biosorpion data was analyzed using Syngistix software; Microscopy data was analyzed using Image J software (version 2.0.0-rc-69/1.52p); Material quantification from images was performed using Ilastik (version 1.3.3) and MATLAB R2020b; Graphs were generated using Origin (2021b);

For manuscripts utilizing custom algorithms or software that are central to the research but not yet described in published literature, software must be made available to editors and reviewers. We strongly encourage code deposition in a community repository (e.g. GitHub). See the Nature Portfolio guidelines for submitting code & software for further information.

Data

Policy information about availability of data

All manuscripts must include a data availability statement. This statement should provide the following information, where applicable:

- Accession codes, unique identifiers, or web links for publicly available datasets
- A description of any restrictions on data availability
- For clinical datasets or third party data, please ensure that the statement adheres to our policy

The datasets generated and/or analysed during the current study have all be submitted as source data files.

Field-specific reporting

Please select the one below that is the best fit for your research. If you are not sure, read the appropriate sections before making your selection.

[x] Life sciences [ ] Behavioural & social sciences [ ] Ecological, evolutionary & environmental sciences

For a reference copy of the document with all sections, see nature.com/documents/nr-reporting-summary-flat.pdf

Life sciences study design

All studies must disclose on these points even when the disclosure is negative.

Sample size	The exact number of samples for each experiment is indicated in the figure caption and in the 'Statistics and Reproducibility' section.
Data exclusions	No data were excluded from analyses
Replication	We state clearly in the legends of every figure and in the added 'Statistics and Reproducibility' section how many times each experiment was repeated independently with similar results. All the independent replicated were successful and showed similar results.
Randomization	All bacterial cell cultures used for each experiments were grown under the same conditions, so randomization was not relevant
Blinding	This study only provides objective measurements of samples derived from bacterial cultures subjected to identical growth conditions per experiment. Blinding was not relevant for this study

Reporting for specific materials, systems and methods

We require information from authors about some types of materials, experimental systems and methods used in many studies. Here, indicate whether each material, system or method listed is relevant to your study. If you are not sure if a list item applies to your research, read the appropriate section before selecting a response.

Materials & experimental systems

n/a	Involved in the study	
[ ]	[x]	Antibodies
[x]	[ ]	Eukaryotic cell lines
[x]	[ ]	Palaeontology and archaeology
[x]	[ ]	Animals and other organisms
[x]	[ ]	Human research participants
[x]	[ ]	Clinical data
[x]	[ ]	Dual use research of concern

Methods

n/a	Involved in the study	
[x]	[ ]	ChIP-seq
[x]	[ ]	Flow cytometry
[x]	[ ]	MRI-based neuroimaging

Antibodies

Antibodies used	Commercial Monoclonal ANTI-FLAG® M2 antibody (Monoclonal ANTI-FLAG® M2-Peroxidase (HRP) antibody - from Sigma Aldrich A8592-.2MG) produced in mouse was used for Immunoblot Analysis of BUD proteins.
Validation	From the Sigma Aldrich website: Monoclonal ANTI-FLAG® M2-Peroxidase is a covalent conjugate of a purified IgG1 monoclonal ANTI-FLAG® M2 antibody, which has been isolated from a mouse cell culture, with horseradish peroxidase (HRP). This antibody-HRP conjugate binds to FLAG® fusion proteins and recognizes the FLAG® epitope at any position in the fusion protein (N-terminal, Met-N-terminal, C-terminal, or internal FLAG® peptides). This conjugate is useful for identification of FLAG® fusion proteins by common immunological procedures. Monoclonal ANTI-FLAG® M2-Peroxidase may be used for the detection of FLAG® fusion proteins. Applications for the conjugate include Western blots, dot blots, ELISA, and immunocytochemistry. Several theses and dissertations cite use of this product in their protocols. Validation of this product can be found here: https://www.sigmaaldrich.com/deepweb/assets/sigmaaldrich/product/documents/144/194/vol6_iss2_antiflag_m2.pdf
