# Supplementary material for: A de novo matrix for macroscopic living materials from bacteria
Source: Nat Commun. 2022 Sep 21;13:5544. doi: 10.1038/s41467-022-33191-2 (PMC9492681; doi:10.1038/s41467-022-33191-2)
Supplement: Supplementary file 4 — Source Data [file 41467_2022_33191_MOESM4_ESM.zip › Source_Data_Files/Source Data_Fig2e.pdf]

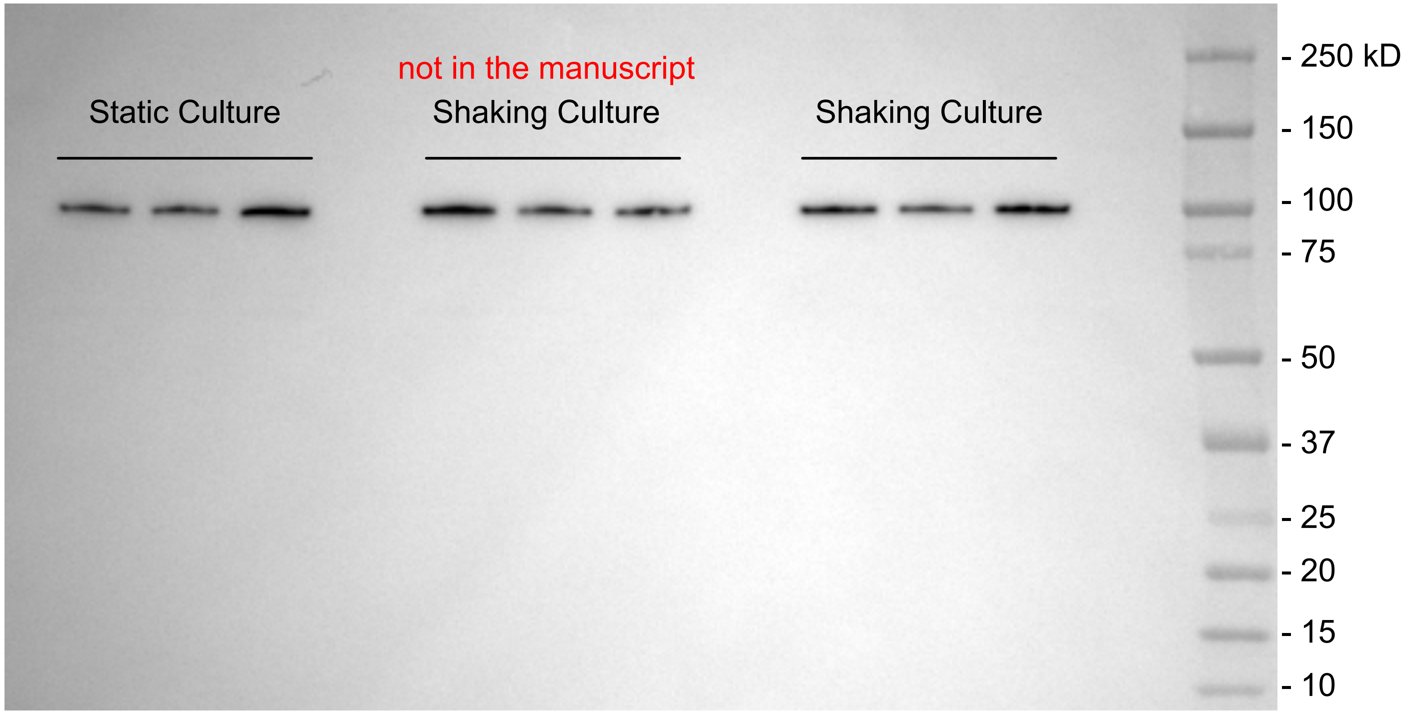
not in the manuscript
Static Culture
Shaking Culture
Shaking Culture
- 250 kD
- 150
- 100
- 75
- 50
- 37
- 25
- 20
- 15
- 10
