# Supplementary material for: A de novo matrix for macroscopic living materials from bacteria
Source: Nat Commun. 2022 Sep 21;13:5544. doi: 10.1038/s41467-022-33191-2 (PMC9492681; doi:10.1038/s41467-022-33191-2)
Supplement: Supplementary file 4 — Source Data [file 41467_2022_33191_MOESM4_ESM.zip › Source_Data_Files/Source Data_Fig2b_ImageAnalysis.rtf]

Procedure Image Analysis
Software: ImageJ (version 2.0.0-rc-69/1.52p)

CELL FLUORESCENCE CHANNEL
Duplicate
Invert
Image—> type—> 32bit
Threshold (mean) - Auto, Apply —> Set to NaN (Background)
Save

MATRIX FLUORESCENCE CHANNEL
Duplicate
Image—> type—> 32bit
Threshold (mean) - Auto, Apply —> Set to NaN (Background)
Save

IMAGE PROCESSING
Open modified Images of both channels
Image Calculator —> cell channel AND matrix channel [click both 32bit float images and open in a new window]
Analyze —> Measure (Set Measurement: Area, Area Fraction)
Save measurements
[this operation gives as results the percentage of overlapping - AND - of the TOTAL image size]

Open modified Images of cell channels
Analyze —> Measure (Set Measurement: Area, Area Fraction)
[this operation gives as results the size of the image minus the cells that were set on NaN by previous operations]

Software: Microsoft Excel 2016
Calculate the pixel of overlapping (with the percentage and the total pixel of the image)
Calculate the percentage of matrix in the non-cell space
